# Unraveling ERBB network dynamics upon betacellulin signaling in pancreatic ductal adenocarcinoma in mice

**DOI:** 10.1002/1878-0261.12699

**Published:** 2020-05-18

**Authors:** Kathrin Hedegger, Hana Algül, Marina Lesina, Andreas Blutke, Roland M. Schmid, Marlon R. Schneider, Maik Dahlhoff

**Affiliations:** ^1^ Institute of Molecular Animal Breeding and Biotechnology Gene Center of the LMU Munich Germany; ^2^ Second Department of Internal Medicine Klinikum rechts der Isar Technical University of Munich Germany; ^3^ Research Unit Analytical Pathology Helmholtz Zentrum München Neuherberg Germany

**Keywords:** BTC, EGFR, ERBB2, ERBB4, mouse model, PDAC

## Abstract

Pancreatic ductal adenocarcinoma (PDAC) will soon belong to the top three cancer killers. The only approved specific PDAC therapy targets the epidermal growth factor receptor (EGFR). Although EGFR is a crucial player in PDAC development, EGFR‐based therapy is disappointing. In this study, we evaluated the role of the EGFR ligand betacellulin (BTC) in PDAC. The expression of BTC was investigated in human pancreatic cancer specimen. Then, we generated a BTC knockout mouse model by CRISPR/Cas9 technology and a BTC overexpression model. Both models were crossed with the *Ptf1a^Cre/+^;KRAS^G12D/+^* (KC) mouse model (B^−/−^KC or BKC, respectively). In addition, EGFR, ERBB2, and ERBB4 were investigated by the pancreas‐specific deletion of each receptor using the Cre‐*loxP* system. Tumor initiation and progression were analyzed in all mouse lines, and the underlying molecular biology of PDAC was investigated at different time points. BTC is expressed in human and murine PDAC. B^−/−^KC mice showed a decelerated PDAC progression, associated with decreased EGFR activation. BKC mice developed severe PDAC with a poor survival rate. The dramatically increased BTC‐mediated tumor burden was EGFR‐dependent, but also ERBB4 and ERBB2 were involved in PDAC development or progression, as depletion of EGFR, ERBB2, or ERBB4 significantly improved the survival rate of BTC‐mediated PDAC. BTC increases PDAC tumor burden dramatically by enhanced RAS activation. EGFR signaling, ERBB2 signaling, and ERBB4 signaling are involved in accelerated PDAC development mediated by BTC indicating that targeting the whole ERBB family, instead of a single receptor, is a promising strategy for the development of future PDAC therapies.

## Graphical abstract

We have shown that BTC overexpression deteriorates pancreatic ductal adenocarcinoma (PDAC) prognosis in mice. BTC enhances RAS activity, thereby potentially transforming acinar to duct cells. BTC involves not only EGFR activation, it also activates ERBB2 and ERBB4 to induce accelerated PDAC development and progression. A partial disease rescue upon loss of ERBB2 and ERBB4 implies that both receptors are potent oncogenes in PDAC.

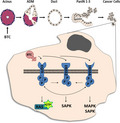

**Abbreviations**
ACTA2: alpha smooth muscle actin
ADM: acinar‐to‐ductal metaplasia
ANOVA: analysis of variance
AREG: amphiregulin
BTC: betacellulinCRISPR/Cas, clustered regularly interspaced short palindromic repeats/CRISPR‐associated
EGF: epidermal growth factor
EGFR: EGF receptor
EREG: epiregulin
HBEGF: heparin‐binding EGF‐like growth factor
hPaCaCells: human pancreatic cancer cell lines
ICD: intracellular domain
IHC: immunohistochemistry
KRAS: Kirsten rat sarcoma viral oncogene homolog
MAPK: mitogen‐activated protein kinase 1/3
PanIN: pancreatic intraepithelial neoplasia
PDAC: pancreatic ductal adenocarcinoma
PFA: paraformaldehyde
*Ptf1a*: pancreas‐specific transcription factor 1 alpha
RIP: regulated intramembrane proteolysis
SAPK: stress‐activated protein kinase
TGFA: transforming growth factor alpha
*Tp53*: tumor protein p53

## 1 Introduction

With a 5‐year survival rate of 8% pancreatic ductal adenocarcinoma (PDAC) is worldwide one of the deadliest cancers. While mortality rates are declining for many cancers due to early detection and improved treatment, rates for PDAC are still rising (Siegel *et al*., 2018), promoting PDAC to the top three cancer killers within the next decade (Rahib *et al*., 2014). Standard PDAC treatment includes surgical resection and adjuvant chemotherapy with FOLFIRINOX or gemcitabine in combination with nab‐paclitaxel providing the most promising results (Aslan *et al*., 2018). However, systemic chemotherapy is associated with severe side effects. There is an urgent need to find customized therapies targeting aberrantly regulated molecules in PDAC. Over 95% of PDAC patients harbor an activating point mutation in the Kirsten rat sarcoma viral oncogene homolog (*KRAS*) gene (Bryant *et al*., 2014). *KRAS^G12D^* is the initiating mutation in PDAC being detected in over 90% of pancreatic intraepithelial neoplasia (PanIN) (Fischer and Wood, 2018). Attempts to target aberrant KRAS in PDAC were promising in preclinical studies. However, their performance in clinical trials was rather disappointing (Zeitouni *et al*., 2016). A further challenge is the limited delivery of therapeutics to the cancer cells in the stroma of PDAC, which has a high extracellular matrix content and shows poor vascularization (Olive, 2015). It was shown that the epidermal growth factor receptor (EGFR), acting upstream of KRAS, was required for oncogenic KRAS‐driven PDAC tumorigenesis in mice with a wild‐type tumor protein 53 (*Tp53*) background (Ardito *et al*., 2012; Navas *et al*., 2012). Indeed, EGFR is the only molecule approved for targeted PDAC therapy in the clinic, albeitwith marginal improvement in survivalwith only a small subset of patients responding. Surprisingly, the response rate to erlotinib in PDAC patients is independent of the pancreatic EGFR expression status (Moore *et al*., 2007). Thus, many questions concerning EGFR signaling in PDAC remain to be addressed. EGFR belongs to the family of the ERBB receptors as ERBB2 (HER2, neu), ERBB3 (HER3), and ERBB4 (HER4). They homo‐ or heterodimerize upon ligand‐dependent activation in order to induce cellular responses like proliferation, migration, apoptosis, differentiation, and adhesion, and with 28 possible receptor combinations (including spliced receptors) and 11 ligands (Schneider and Wolf, 2009), the family is able to induce 611 different active receptor/ligand combinations (Roskoski, 2014). Many ERBB ligands like EGF and transforming growth factor alpha (TGFA) (Wagner *et al*., 1998), amphiregulin (AREG) (Wang *et al*., 2016), heparin‐binding EGF‐like growth factor (HBEGF) (Ray *et al*., 2014), and epiregulin (EREG) (Zhu *et al*., 2000) have been associated with PDAC. While all of them bind EGFR, a subset also binds ERBB4, indicating that EGFR might not be the only candidate to mediate their effects in PDAC. A further EGFR‐ and ERBB4‐binding ligand, betacellulin (BTC) has also been associated with PDAC. *BTC* mRNA was detected in human pancreatic cancer cell lines (hPaCaCells) and elevated in human PDAC tissues (Yokoyama *et al*., 1995). BTC was also revealed to be a potent mitogen in hPaCaCells, while the transmitting receptors remained unidentified (Kawaguchi *et al*., 2000). This is particularly interesting since its designated receptor ERBB4 plays controversial roles in PDAC development and progression (Graber *et al*., 1999; Kolb *et al*., 2007; Mill *et al*., 2011). The role of ERBB4 seems to be context‐dependent, probably due to its—in contrast to its ERBB relatives—ability to signal in form of its soluble intracellular domain (ICD80) after undergoing regulated intramembrane proteolysis (RIP) (Carpenter, 2003), induced by tumor necrosis factor alpha converting enzyme (Kenny and Bissell, 2007). However, in another pancreatic disorder BTC transgenic mice were protected against acute pancreatitis mediated by ERBB4 signaling and independent of EGFR (Hedegger *et al*., 2019). This demonstrates the complexity of the ERBB system and points out the versatile responses to ERBB ligands in a tissue‐specific manner. There is an urgent need to unravel the intricate ERBB network in pancreatic cancer to better understand not only the role of EGFR, but also to show the potential significance of its relatives in order to establish more efficiently targeted therapies. To investigate BTC in pancreatic cancer, we generated a BTC knockout mouse model (BTC^−/−^) and overexpressed BTC in a transgenic mouse model (*Btc^tg/+^*). Both models were crossed in a PDAC mouse model, and to assess the receptor dependency, BTC‐transgenic mice were also crossed with PDAC mouse lines with pancreas‐specific EGFR, ERBB2, or ERBB4 deletions.

## 2 Materials and methods

### 2.1 Human samples

For ERBB receptor immunohistochemistry (IHC), primary PDACs that were resected between 2008 and 2013 at the Klinikum rechts der Isar, Technische Universität München, were used after written informed consent was obtained. Pancreatic samples from nondiseased pancreas served as controls. The use of this patient cohort for biomarker analysis has been approved by the ethics commission of the Klinikum Rechts der Isar, Technische Universität München (403/17S), and the study methodologies conformed to the standards set by the Declaration of Helsinki.

### 2.2 Animals

All animal experiments were approved by the author’s institutional committee on animal care and carried out in accordance with the German Animal Protection Law with permission from the responsible veterinary authority (Az.:55.2‐1‐54‐2532‐26‐2014). Mice carrying floxed *Egfr^fl/fl^* (*Egfr^tm1Dwt^*) (Lee and Threadgill, 2009), *Erbb2^fl/fl^(*Garratt *et al*., 2000 *)*, *Erbb4^fl/fl^* (*B6;129‐Erbb4^tm1Fej/Mmucd^*) *(*Long *et al*., 2003 *)*, and *Kras^G12D/+^* (*B6.129S4‐Kras^tm4Tyj^/J*)(Jackson *et al*., 2001) alleles or expressing Cre recombinase under the pancreas‐specific transcription factor 1 alpha (*Ptf1a*‐Cre) (*Ptf1a^tm1(cre)Hnak^*) (Nakhai *et al*., 2007) promoter have been described previously. Transgenic mouse lines ubiquitously overexpressing BTC under the control of the chicken‐beta‐actin gene promoter (*Btc^tg/+^*) have been described elsewhere (Schneider *et al*., 2005). We cross‐mated *Btc^tg/+^;Ptf1a^Cre/+^;Kras^G12D/+^* (herein referred to as BKC) mice and *Btc^tg/+^;Ptf1a^Cre/+^;Kras^G12D/+^;Egfr^fl/fl^*, *Btc^tg/+^;Ptf1a^Cre/+^;Kras^G12D/+^;Erbb2^fl/fl^*, and *Btc^tg/+^;Ptf1a^Cre/+^;Kras^G12D/+^;Erbb4^fl/fl^* mice (herein referred to as E1KO;BKC, E2KO;BKC, and E4KO;BKC, respectively) to delete the designated ERBB receptor pancreas‐specifically using the Cre‐*loxP*‐system. The genotypes of all mice were verified by PCR (Qiagen, Hilden, Germany), employing genomic DNA from tail tips by using the oligonucleotides listed in Table S1. Mice were maintained in the C57BL/6N background and housed under specific pathogen‐free conditions in the closed barrier facility of the Gene Center Munich at 23 °C, 50% humidity, and with a 12‐h light/dark cycle (lights on at 7 AM). They had free access to water and a standard rodent diet (V1534, Ssniff, Soest, Germany). Mice were weighed weekly until the age of 6 months and afterwards, still weekly or every 2 weeks. Mice were killed at the designated time points or, for survival analysis, were left alive and killed as soon as they became moribund.

### 2.3 **Generation of BTC knockout mice (BTC** ^−/−^ **)**

For CRISPR/Cas9‐assisted *Btc* gene disruption using a single guide RNA (sgRNA) specific for exon 2 sequence 5′‐GTCTTGCAATTCTCCACTGTG‐3′, a corresponding oligonucleotide was cloned into the pEX‐A‐U6‐gRNA vector as described previously (Dahlhoff *et al*., 2017). Cas9 mRNA and sgRNA were *in vitro*‐transcribed by using the Ambion Maxiscript SP6 kit (Thermo Fisher Scientific, Waltham, MA, USA). C57BL/6N zygotes were injected with Cas9 mRNA (50 ng·µL^−1^) and sgRNA (100 ng·µL^−1^), and embryos were transferred into recipient NMRI mice. Potential founders were identified by PCR using the primers listed in Table S1. Based on the detected mutations, a *PfiM*I restriction fragment length polymorphism assay was established, yielding fragments of 210 and 290 bp for wild‐type *Btc* and a single fragment of 500 bp for the mutated *Btc* sequence. Two founder animals with monoallelic insertions of 1 bp were identified. The insertion of 1 bp leads to a shift in the reading frame in *Btc* exon 2. The mutated *Btc* transcripts encode 27 amino acids (aa) of the extracellular BTC domain, followed by a 31 aa missense sequence and a premature termination codon after 58 aa (Fig. S1).

### 2.4 Pancreas preparation

Mice were sacrificed by cervical dislocation at the age of 1 week, 8 weeks, 12 months, or when moribund. The pancreas was isolated, blotted dry, and weighed to the nearest milligram. Parts of the head, tail, and central part of the pancreas were dissected, pooled, frozen on dry ice, and stored at −80 °C. The remaining tissue was fixed in 4% para‐formaldehyde (PFA, in PBS, pH 7.4) overnight and subsequently embedded in paraffin for histopathological examination. From mice sacrificed at the age of 1 week, the whole pancreas was either frozen on dry ice or immediately homogenized in total in RLT buffer (Qiagen), freshly supplemented with 1% beta‐mercaptoethanol (Roth, Karlsruhe, Germany) and shock‐frozen in liquid nitrogen for RNA isolation, or incubated as a whole in 4% PFA overnight and embedded in paraffin.

### 2.5 Immunohistochemistry

Immunohistochemistry was performed, using specific antibodies for detection of murine (m) mBTC, mTGFA, mAREG, mEREG, mACTA2, mEGFR, cleaved caspase‐3, and human (h) hBTC, hEGFR, hERBB2, hERBB3, and hERBB4 in sections of PFA‐fixed, paraffin‐embedded pancreatic tissue. For all immunostainings, the slides were boiled in a pressure cooker for 15 min in 10 mm sodium citrate buffer pH 6.0 or EDTA pH 9, blocked in 3% H_2_O_2_ for 30 min and in 5% of the appropriate serum for another 30 min. Primary antibodies were incubated over night at 4 °C. After washing in PBS, the slides were incubated with the appropriate secondary antibodies for 1 h at room temperature, and the signal was amplified using the VECTASTAIN® ABC HRP kit (Vector, Burlingame, CA, USA) for 30 min at room temperature. As chromogen, ImmPACT^TM^ DAB peroxidase substrate kit (Vector) was used and the sections were counterstained with hematoxylin (Roth) for 3 min. A list of primary and secondary antibodies with the corresponding dilutions is provided in Table S2. Appropriate negative control sections (omission of the first antibodies) were carried along in all IHC experiments.

### 2.6 Histopathology and morphometric analyses

For histological analyses, the PFA‐fixed and paraffin‐embedded pancreas was serially sectioned and four sections with a distance of nine sections between were sampled and stained with hematoxylin and eosin (H&E), and Masson’s trichrome, respectively. The sections were independently analyzed by two researchers in a blinded fashion. (Pre‐) neoplastic pancreas alterations were classified, using established histomorphological criteria (Distler *et al*., 2014; Hruban *et al*., 2004). For quantification of lesions, the relative section areas of altered tissue in the pancreas were determined. The fractional area of the total ‘reactive tissue’ (comprising fibrosis, inflammation, preneoplastic lesions, including acinar‐to‐ductal metaplasia (ADM) and PanIN of grades 1‐3, as well as PDAC) in the pancreas was quantified in digital images covering the complete area of all pancreas sections (200× magnification) of *n* = 4 mice of 8 weeks of age per group, using las software version 3.8.0 (Leica Microsystems, Wetzlar, Germany).

Additionally, the area density of acinar cell section profiles, ADM, PanIN1‐3, PDAC, and fibrosis in the pancreas of 12‐month‐old mice (*n* = 4/group) was separately determined by point counting (Howard and Reed, 2004; Weibel, 1979). For this, digital images of H&E or Masson’s trichrome‐stained sections were superimposed with a grid of equally spaced crosses (117 crosses/2 cm^2^), using netscope viewer software (Net‐Base Software GmbH, Freiburg, Germany). Crosses hitting section profiles of the respective structure were counted and related to the number of crosses hitting pancreas tissue in all examined sections per case. On the average, 111 ± 7 points were counted per case. Data were analyzed by Student’s *t*‐test, respectively, by 2‐way ANOVA and plotted as column bar plots in graphpad prism (GraphPad Prism version 5.0 for Windows, GraphPad Software, San Diego, CA, USA).

### 2.7 RAS activity assay

To evaluate pancreatic RAS activity, the Active Ras Detection Kit (Cell Signaling, Frankfurt, Germany) was used according to the manufacturer’s instructions. In brief, dissected, frozen pancreas was homogenized in lysis/binding/wash buffer, freshly supplemented with phenylmethanesulfonyl fluoride, and 300 µg total protein was incubated with the GST‐Raf1‐Ras‐binding domain for 1 h at 4 °C, washed and eluted under denaturing conditions and applied to an SDS‐gel electrophoresis using Mini‐PROTEAN® TGX Stain‐Free™ Precast Gels (BIO‐RAD, Hercules, CA, USA) and subsequent Western blot analysis detecting mRAS. Total protein was quantified using Image Lab 6.0.1 (Bio‐Rad), and the amount of active RAS was referenced to total protein and plotted in graphpad prism. Data were analyzed by Student’s *t*‐test.

### 2.8 Co‐immunoprecipitation

Pancreata were homogenized in a TRIS‐based buffer (50 mm Tris, 150 mm NaCl, 1% NP‐40, 10% glycerol, 1 m EDTA; freshly supplemented with protease, and phosphatase inhibitors), and 350 µg of protein was immunoprecipitated by targeting EGFR. For this, the lysate was incubated with 1 µg of EGFR antibody (Santa Cruz, SC‐03, Heidelberg, Germany) or 1 µg of normal IgG antibody (R&D Systems, Minneapolis, MN, USA) at 4 °C overnight and incubated with 50 µL of protein A‐coated magnetic beads (Cell Signaling) for 30 min at room temperature. After washing, the precipitate was eluted under denaturing conditions and applied to a western blot detecting ERBB2 (Santa Cruz, SC‐284), ERBB3 (Santa Cruz, SC‐285), and EGFR as control.

### 2.9 Western blot analysis

Pancreas of animals was homogenized in Laemmli‐extraction buffer as described previously (Dahlhoff *et al*., 2015). Protein samples with equal concentrations were electrophoresed on 10% polyacrylamide–sodium dodecyl sulfate gels and blotted to polyvinylidene difluoride membranes (GE Healthcare, Munich, Germany). The membranes were blocked with 5% milk and incubated with the primary antibodies overnight at 4 °C. After washing, the membranes were incubated in the appropriate horseradish peroxidase‐conjugated secondary antibody. Immunoreactive bands were visualized by chemiluminescence with an ECL Kit (GE Healthcare or Thermo Scientific). Antibodies and dilutions are supplied in Table S3. Densitometrical analyses were performed with imagej 1.52a (http://rsb.info.nih.gov/ij) and plotted in graphpad prism (GraphPad Prism version 5.0).

### 2.10 Reverse transcriptase–PCR

RNA was extracted from different organs with TRIzol reagent (Invitrogen, Darmstadt, Germany), and 3 µg of RNA was reverse‐transcribed in a final volume of 30 µL using RevertAid reverse transcriptase (Thermo Scientific, Schwerte, Germany) according to the manufacturer’s instructions. To show the qualitative mRNA expression of Btc‐KO mice, reverse transcription–PCR (RT–PCR) was performed by using reagents from Qiagen. The final reaction volume was 20 µL, and cycle conditions were 94 °C for 5 min, followed by 35 cycles of 94 °C for 1 min, 58 °C for 1 min, and 72 °C for 1 min. The amplicon for *Btc* was digested by *PflM*I (NEB, Frankfurt, Germany) for 90 min at 37 °C and subject to agarose electrophoresis to reveal the mutation side. *Gapdh* was used as reference mRNA. The used primers are listed in Table S1.

### 2.11 Hematoxylin and eosin (H&E) and Masson’s trichrome staining

H&E‐stainings (histological standard stain) and Masson’s trichrome stainings (demonstration of collagenous connective tissue) were performed on sections of PFA‐fixed and paraffin‐embedded pancreas tissue, using standard protocols.

### 2.12 Cell culture and stimulation experiments

PANC‐1 and BcPC‐3 cells were purchased from CLS (Cell Lines Service, Eppelheim, Germany) 4 months before the experiments were performed. All human permanent cell lines in the CLS cell bank were authenticated by using the STR DNA profiling analysis. Mycoplasma testing was done every 6 months for all cultured cells, using a Mycoplasma Detection Kit (PlasmoTest; InvivoGen, Toulouse, France). Both cancer cell lines were maintained at 37 °C and 5% CO_2_. PANC‐1 cells were cultured in Dulbecco’s modified Eagle Medium (Merck, Darmstadt, Germany), BxPC‐3 cells in Roswell Park Memorial Institute 1640 medium (RPMI; Merck), both supplemented with 10% FBS (Merck) and 1% Penicillin/Streptomycin (Merck). At a confluence of 90%, cells were starved overnight (1% FBS) and stimulated the next day with 50 ng·mL^−1^ of recombinant human BTC (rhBTC; R&D Systems #261‐CE) for 5 and 15 min. Cells were then lysed in a Tris‐based buffer [50 mm Tris, 150 mm NaCl, 1% NP‐40, 10% glycerol, 1 m EDTA; freshly supplemented with protease and phosphatase inhibitors (Roche, Penzberg, Germany)] and subject to western blot analysis detecting phosphorylation and total expression of the ERBB receptors.

### 2.13 3D primary cell culture

3D primary cell cultures of pancreatic acini were prepared according to a modified protocol of Qu and Konieczny (2013). For isolation of acini, freshly dissected pancreata of 3‐week‐old wild‐type mice were washed twice in sterile ice‐cold PBS and immediately minced and digested twice in collagenase P solution (Hanks Balanced Salt Solution; Sigma, Taufkirchen, Germany), 5% FBS, 0.2 mg·mL^−1^ soybean trypsin inhibitor (STI; Sigma), 0.2 mg·mL^−1^ Collagenase P (Roche) at 37 °C for 10 min. The pancreatic tissue was gently pressed and washed through a 100‐µm cell strainer and incubated in red blood cell lysis buffer (Roth) for 10 min at 37 °C. The acini recovered for 1 h in 3D culture medium [RPMI 1640, 1% FBS, 1% Penicillin/Streptomycin, 1 mg·mL^−1^ STI, 1 µg·mL^−1^ dexamethasone (Sigma)]. Prior to cell seeding, the culture dishes were coated with a matrix of rat tail collagen I (Invitrogen, Carlsbad, CA, USA) and RPMI medium, supplemented with NaHCO_3_ for at least 1 h at 37 °C. For seeding, the cell suspension was mixed with the collagen I coating gel in a ratio 1 : 3, gently placed into the coated dishes, and solidified for 1 h at 37 °C, 5% CO_2_. The matrix was then coated with warm 3D culture medium and stimulated or left untreated. For stimulation experiments, 3D culture medium was supplemented with rhBTC or rhTGFA or left untreated and investigated under a light microscope at days 0, 4, and 5 after treatment. The number of transdifferentiated cells was estimated by two researchers, independently.

### 2.14 Statistics

Data are presented as means ± SEM and compared by two‐tailed unpaired Student’s *t*‐test, and in the case of more than two groups by analysis of variance (ANOVA) and Tukey’s multiple comparison test. All data were analyzed with graphpad prism (GraphPad Prism version 5.0 for Windows, GraphPad Software, San Diego, CA, USA). *P*‐values < 0.05 were considered statistically significant.

## 3 Results

### 3.1 BTC and the ERBB receptors are expressed in human pancreas, PDAC samples, PDAC cell lines, and in pancreata of PDAC mice

**Fig. 1:**
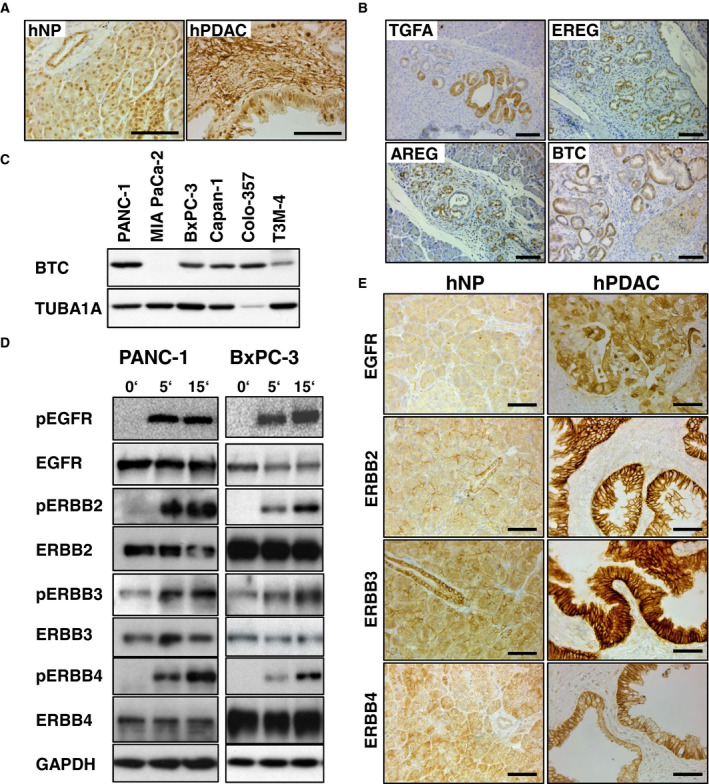
BTC and the ERBBs are expressed in human pancreas, PDAC samples, in human PDAC cell lines, and in pancreata of KC mice. (A) Immunohistochemical detection of BTC in hNP and in human PDAC specimen. (B) Immunohistochemical detection of BTC, TGFA, AREG, and EREG in PanIN of KC mice. Scale bars in A and B: 100 µm. (C) Western blot analysis of BTC expression in hPaCaCells. TUBA1A served as a reference protein. (D) Western blot analysis of ERBB receptor expression and phosphorylation in PANC‐1 cells and BxPC‐3 cells after BTC stimulation. GAPDH served as reference protein. (E) IHC of all ERBB receptors on hNP and hPDAC. Scale bars: 50 µm.

Betacellulin was detected by IHC predominantly in the islets of Langerhans and in ducts and acini of normal human pancreas (hNP), in all examined human PDAC samples (6/6), in cancer cells, and the adjacent stroma (Fig. 1A). Western blot analysis revealed BTC expression in 5/6 hPaCaCells (Fig. 1C). Furthermore, all ERBB receptors were detected by IHC in human PDAC (Fig. 1E). Since BTC can activate all ERBB receptors, either directly or indirectly, we evaluated the phosphorylation status of all ERBB receptors upon BTC stimulation in two hPaCaCells. Stimulation of PANC‐1 (with *KRAS* mutation) and BxPC‐3 (with wild‐type*KRAS*) cells with hBTC led to the activation of all receptors at 5 and 15 min, while ERBB3 was constitutively activated in both cell lines (Fig. 1D). These data indicate that BTC and the ERBB receptors are associated with human PDAC. The pancreata of *Ptf1a^c^^re/+^;Kras^G12D/+^* (herein referred to as KC) mice revealed a strong positive immunostaining for endogenous BTC expression in low‐ and high‐grade PanIN in 5/6 samples (Fig. 1B).

### 3.2 Lack of BTC in KC mice results in a reduction of tumor burden and decreased EGFR phosphorylation

**Fig. 2:**
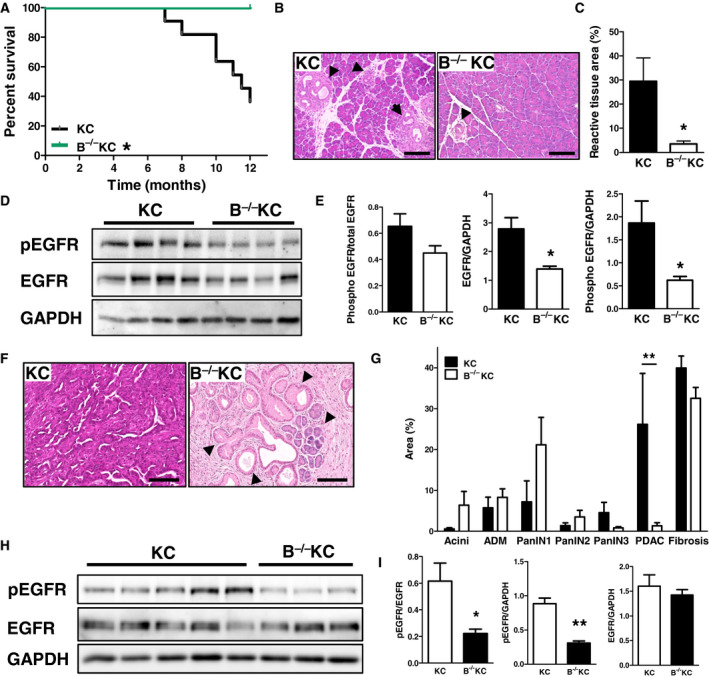
Characterization of B^−/−^KC mice. (A) Kaplan–Meier curve demonstrating the survival of KC and BKC mice. (B) Representative H&E stainings of pancreata of 8‐week‐old B^−/−^KC mice compared to age‐matched KC littermates, typical lesions are indicated by arrowheads. (C) Morphometric analysis of reactive tissue in the pancreas of 8‐week‐old B^−/−^KC and KC mice. Data were analyzed by Student’s *t*‐test. (D) Western blot analysis and (E) corresponding densitometrical analyses showing EGFR expression and phosphorylation in pancreata of 8‐week‐old B^−/−^KC mice compared to age‐matched KC pancreata. GAPDH served as reference protein. (F) Representative H&E stainings of pancreata of 12‐month‐old mice, typical lesions are indicated by arrowheads. (G) Histopathological grading of pancreata of 12‐month‐old mice. (H) Western blot and (I) densitometrical analysis of pancreata of 12‐month‐old mice showing EGFR expression and phosphorylation in B^−/−^KC mice compared to KC mice. Scale bars: 100 µm. Data are presented as means ± SEM. *P < 0.05, **P < 0.01.

To evaluate the function of BTC in PDAC development, we generated a BTC knockout mouse (BTC^−/−^) by CRISPR/Cas9 (clustered regularly interspaced short palindromic repeats/CRISPR‐associated 9) technology (Fig. S1A). BTC^−/−^ mice were viable and showed no macroscopic phenotype, and bred in a Mendelian ratio (data not shown). RT–PCR analysis confirmed the frameshift in exon 2 of the *Btc* gene (Fig. S1B), and the loss of BTC was confirmed by IHC (Fig. S1D). Crossed into the KC background (herein referred to as B^−/−^KC mice), B^−/−^KC mice showed no differences in body and relative pancreas weight at the age of 8 weeks and 12 months, respectively (Fig. S1C). All B^−/−^KC animals remained clinically unremarkable to the time of dissection at 12 months, while KC mice started deceasing already at 7 months (Fig. 2A). The fractional section area of reactive tissue (fibrosis, inflammation, ADM, PanIN) in the pancreas was determined in 8‐week‐old mice. At this age, B^−/−^KC mice showed a significant 10‐fold decrease in area densities of reactive tissue in the pancreas as compared to KC mice (Fig. 2B,C), indicating a decelerated PDAC development. In pancreata of 12‐month‐old mice, we observed decreased PDAC progression upon BTC depletion. Compared to KC mice, B^−/−^KC mice displayed higher area densities of preneoplastic lesions (ADM, PanIN1‐2) in the pancreas, whereas the fractional section areas occupied by PDAC were significantly decreased (Fig. 2F,G). Western blot analysis revealed reduced EGFR expression and phosphorylation in pancreata of 8‐week‐old (Fig. 2D,E) and reduced EGFR phosphorylation in 12‐month‐old (Fig. 2H,I) B^−/−^KC mice compared to age‐matched KC mice. These data indicate that the depletion of BTC attenuates tumor initiation and progression by downregulating EGFR signaling, which results in a prolonged survival.

### 3.3 Overexpression of BTC in KC mice leads to early onset of PDAC and a high mortality

**Fig. 3:**
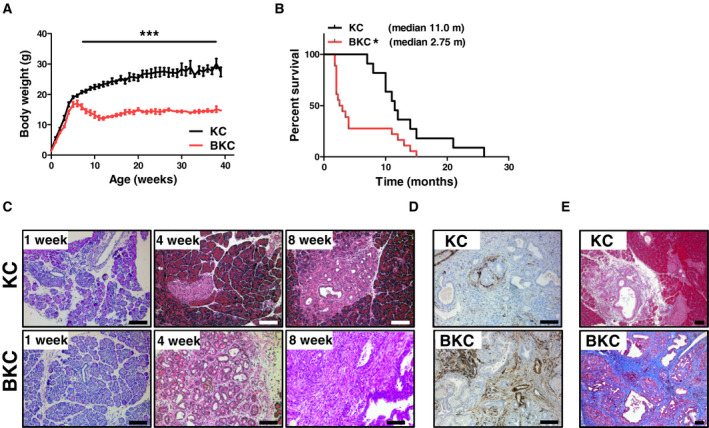
BTC induces adverse events in KC mice. (A) Body weight curve of BKC mice compared to KC littermates. Data were analyzed by 2‐way ANOVA. (B) Kaplan–Meier curve depicting the survival of BKC mice compared to KC mice. Data were analyzed using a log‐rank test. (C) H&E staining of pancreata of 1‐, 4‐ and 8‐week‐old KC and BKC mice. (D) Representative immunohistochemical detection of ACTA2 in pancreata of 8‐week‐old KC and BKC mice. (E) Masson’s trichrome staining of pancreata of 8‐week‐old KC and BKC mice depicting connective tissue in BKC mice. Scale bars: 100 µm. Data are presented as means ± SEM. *P < 0.05, ***P < 0.001.

To investigate how BTC influences PDAC, we overexpressed BTC in a murine PDAC model. We crossed ubiquitously overexpressing BTC mice into the KC background (*Ptf1a^c^^re/+^;Kras^G12D/+^;Btc^tg/+^*, herein referred to as BKC mice). BKC mice developed cachexia after 6 weeks and lost up to 25% of their body weight within the following 2 weeks (Fig. 3A). The major cohort of BKC mice (13/18) was dead at 2 months (median survival: 2.75 months), whereas KC mice had a median survival of 11 months (Fig. 3B). H&E staining of pancreata of 1‐week‐old mice of both groups appeared normal (Fig. 3C). Already at the age of 4 weeks, up to two thirds of the BKC pancreata were covered by inflammation, fibrosis, ADM, and low‐grade PanIN, while these structural abnormalities were rarely observed in age‐matched KC pancreata (Fig. 3C). At 8 weeks of age, reactive lesions occupied only a minor portion of pancreas sections in KC mice (Fig. 3C). In contrast, the pancreas parenchyma of age‐matched BKC mice was consistently replaced completely by low‐ and high‐grade PanIN and invasive carcinoma accompanied by a marked desmoplastic reaction. Desmoplasia was indicated by alpha‐smooth‐muscle‐actin (ACTA2) staining (Fig. 3D), detecting activated pancreatic stellate cells and by Masson’s trichrome staining revealing massive amounts of collagen fibers in BKC animals compared to age‐matched KC mice (Fig. 3E). 75% of KC mice developed high‐grade PanIN and carcinoma during their lifetime, while all BKC mice had developed carcinomas already at the age of 8 weeks.

### 3.4 BTC activates ERBB receptors, enhances RAS activity, and induces ADM

**Fig. 4:**
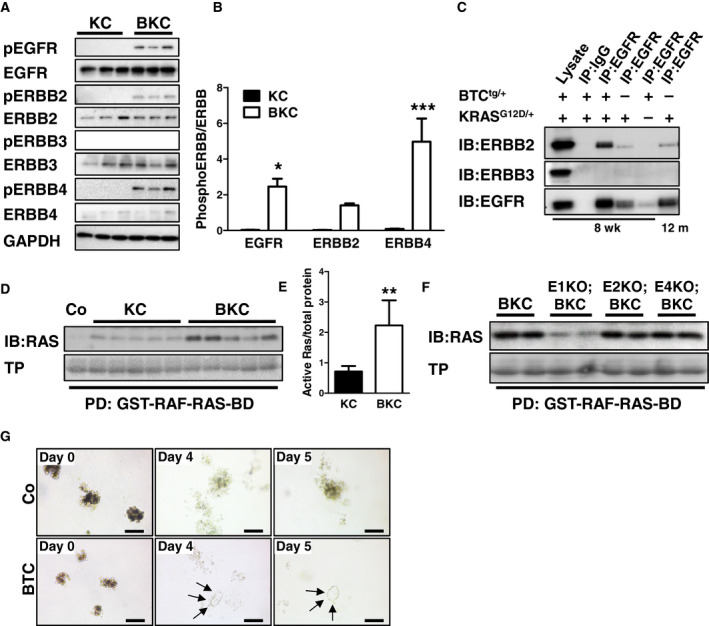
BTC activates ERBB receptors, enhances RAS activity, and induces ADM. (A) Western blot and (B) corresponding densitometrical analysis showing the phosphorylation of EGFR, ERBB2, ERBB3, and ERBB4 in pancreata of 1‐week‐old BKC mice compared to age‐matched KC mice. GAPDH served as reference protein. Data were analyzed by 2‐way ANOVA with Bonferroni post‐test. (C) Co‐immunoprecipitation of EGFR and indicated receptors in lysates of pancreata of 8‐week‐old and 12‐month‐old mice with different genotypes. (D) RAS activity assay with (E) corresponding densitometrical analysis illustrating the abundance of active RAS protein in pancreata of KC mice upon BTC overexpression. Total protein (TP) served as reference. Data were analyzed with Student‘s *t*‐test. (F) RAS activity assay comparing pancreata of BKC, E1KO;BKC, E2KO;BKC, and E4KO;BKC mice. (G) 3D primary tissue culture of acinar cells of 3‐week‐old wild‐type mice, treated with BTC compared to untreated cells (Co). Arrowheads indicate duct formation. Scale bars: 100 µm. PD, pull down, IB, immunoblot, GST‐RAF‐RAS‐BD, glutathione‐*S*‐transferase‐RAF‐RAS‐binding domain. Data are presented as means±‐SEM. *P < 0.05, **P < 0.01, ***P < 0.001.

We investigated ERBB receptor phosphorylation by Western blot analysis upon BTC overexpression in KC mice at the age of 1 week, when pancreatic tissue sections of KC mice did not yet exhibit histomorphological evidence of ADM alterations and displayed a homogenous cellular composition. BTC overexpression in KC mice resulted in the activation of EGFR, ERBB2, and ERBB4. ERBB3 phosphorylation was not detected in both groups. ERBB expression levels were similar in both groups (Fig. 4A,B). To evaluate the receptor dimerization behavior upon BTC activation, we performed co‐immunoprecipitations. In BKC mice, EGFR bound ERBB2, but not ERBB3. KC mice had much less EGFR/ERBB2 dimers, even when the reduced amount of pulled‐down EGFR protein was considered. Due to the high heterogeneity of tissue composition in pancreata of BKC mice at the age of 8 weeks, we compared tumors of 8‐week‐old BKC mice with tumors of 12‐month‐old KC mice, which presented a similar tumor burden. The amount of EGFR/ERBB2 dimers in KC pancreata was again lower compared to BKC mice (Fig. 4C). ERBB3 did not bind to EGFR at any age. There are several ways for BTC to accelerate PDAC development. KRAS^G12D^ is the initiating mutation in PDAC, but the latency for tumor development is very long, and often additional stimuli are necessary to induce RAS‐dependent transformation of normal tissue (Carriere *et al*., 2009; Hingorani *et al*., 2003; Ji *et al*., 2009). We assumed that BTC could be a driver of RAS activity to accelerate tumor development. A RAS‐activity assay of pancreata of 8‐week‐old KC mice and BKC mice revealed that BKC mice harbor a significantly higher amount (threefold) of active RAS compared to KC mice (Fig. 4D,E). To investigate which receptors transmit BTC‐induced RAS activation, we studied RAS activity in the pancreata of BKC mice with a depletion of either EGFR (E1KO;BKC), ERBB2 (E2KO;BKC), or ERBB4 (E4KO;BKC). Notably, BTC‐mediated RAS activity was exclusively transmitted by EGFR (Fig. 4F). Also crucial for PDAC is ADM development. Since it is known that BTC regulates (trans‐) differentiation in numerous cells (Li *et al*., 2005; Paz *et al*., 2011; Yoshida *et al*., 2002), we assumed that BTC might be involved in transdifferentiating acinar to duct cells, thereby promoting the accelerated onset of PDAC observed in BKC mice. We isolated wild‐type murine acinar cells, embedded them into a 3D collagen matrix and stimulated with BTC or TGFA for 5 days. While wild‐type cell clusters did not show signs of transformation, the majority of BTC‐stimulated cells transformed into a duct‐like shape (Fig. 4G).

### 3.5 ERBB2 and ERBB4 affect the tumor burden in BKC mice

**Fig. 5:**
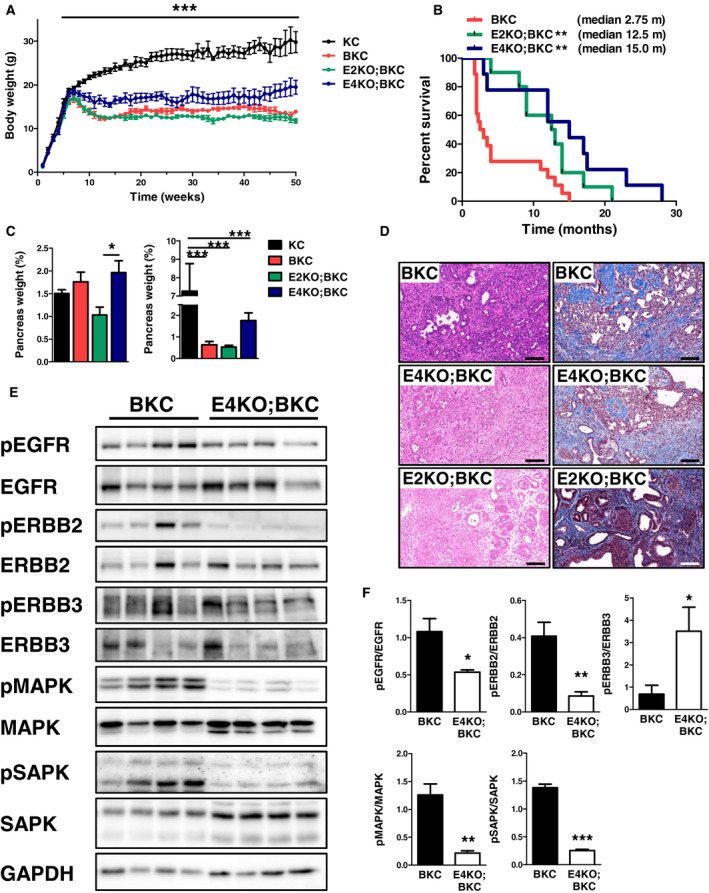
ERBB2 and ERBB4 affect the tumor burden in BKC mice. (A) Body weight curves all groups. Data were analyzed by 2‐way ANOVA. (B) Kaplan–Meier curve depicting the survival of E2 KO;BKC and E4 KO;BKC mice compared to BKC mice. Data were analyzed by log‐rank test. (C) Relative pancreatic weight of all groups of 8‐week‐old mice (left panel) and of 12‐month‐old mice (right panel) compared to E2KO;BKC, E4KO;BKC, and KC mice. Data were analyzed by ANOVA and Tukey’s multiple comparison tests. (D) Histology of BKC mice compared to E2KO;BKC and E4KO;BKC mice at 8 weeks represented by H&E (left panel) and Masson’s trichrome staining (right panel). (E) Western blot analysis with (F) corresponding densitometrical analysis comparing pancreata of 8‐week‐old BKC mice to E4KO;BKC littermates. Data were analyzed by ANOVA and Tukey’s multiple comparison tests. *P < 0.05, **P < 0.01, ***P < 0.001. Scale bars: 100 µm.

Betacellulin signaling is mediated by EGFR‐activated RAS activity, but it remains unknown whether ERBB2 and ERBB4 are involved in BTC‐mediated development of PDAC. We therefore generated BKC mice with a pancreas‐specific knockout of ERBB2 or of ERBB4 (E2KO;BKC and E4KO;BKC, respectively). The lack of pancreatic ERBB2 or ERBB4 expression in the BKC mouse showed distinct effects: While all groups grew similarly until week 6 after birth, E2KO;BKC mice had a significantly reduced body weight compared to BKC mice, and E4KO;BKC mice showed a significantly increased body weight compared to BKC littermates (Fig. 5A), predicting a better outcome for mice with an ERBB4 depletion. Indeed, the Kaplan–Meier curve depicts, with a median survival of 15 months, the longest survival in E4KO;BKC mice. Also E2KO;BKC mice present—with a median survival of 12.5 months—a significantly prolonged survival compared to BKC mice (median survival: 2.75 months) (Fig. 5B), indicating oncogenic functions for both receptors. The relative pancreatic weight of E2KO;BKC mice was decreased compared to that of BKC mice at the age of 8 weeks (*P* = 0.059) (Fig. 5C, left panel) and was similarly lower in moribund mice (Fig. 5C, right panel). The H&E (Fig. 5D, left panel) and Masson’s trichrome (Fig. 5D, right panel) staining of E4KO;BKC pancreata revealed a penetration of cancer cells similar to BKC mice, presenting with fibrosis, PanIN, and carcinoma, while the lack of ERBB2 rather resembled atypical flat lesions mixed with ADM and low‐grade PanIN, with an equal amount of fibrosis in BKC pancreata, but with decreased area of (pre‐)neoplastic tissue, and remaining acinar tissue was frequently observed. Western blot analysis revealed that the loss of ERBB4‐induced changes in the ERBB signaling network (Fig. 5E,F). The lack of ERBB4 resulted in significantly decreased EGFR and ERBB2 phosphorylation, and it induced enhanced ERBB3 activation. Further, the lack of ERBB4 resulted in a significant downregulation of mitogen‐activated protein kinase 1/3 (MAPK) and stress‐activated protein kinase (SAPK) signaling. In 1‐week‐old mice, EGFR activation was significantly enhanced in E4KO;BKC pancreata, and also MAPK activation was increased (Fig. S2A,B). Compared to the knockout of ERBB4, the lack of ERBB2 only resulted in significantly decreased SAPK signaling and in decreased EGFR phosphorylation (*P* = 0.055). However, no significant compensation of other ERBB receptors was observed (Fig. S3A,B). Despite decreased SAPK signaling, acinar cells of E2KO;BKC mice demonstrated cleaved caspase‐3 positivity in immunostainings (Fig. S3C).

### 3.6 The loss of EGFR almost fully rescues the BTC‐mediated phenotype in 8‐week‐old KC mice

**Fig. 6:**
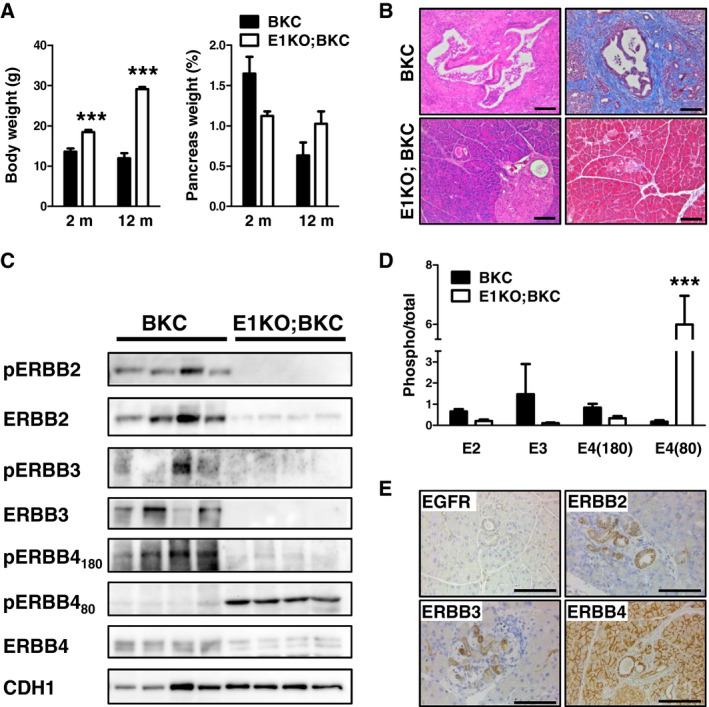
The lack of EGFR in BKC mice revealed major changes in body weight, histology, and ERBB signaling. (A) Body and relative pancreas weights of E1KO;BKC mice compared to BKC mice. Data were analyzed by ANOVA and Tukey’s multiple comparison test. (B) Representative H&E (left panel) and Masson`s trichrome (right panel) stainings of pancreata of BKC mice and E1KO;BKC mice. (C) Western blot analysis and (D) corresponding densitometrical analysis showing the phosphorylation and expression of ERBB2, ERBB3, and full‐length ERBB4, and the phosphorylation of the ERBB4‐ICD. CDH1 served as reference protein. Data were analyzed by ANOVA and Tukey’s multiple comparison test. (E) Immunohistochemical detection of EGFR, ERBB2, ERBB3, and ERBB4 of pancreas samples of 8‐week‐old E1KO;BKC mice. Scale bars: 100 µm. ***P < 0.001.

We deleted EGFR (E1) specifically in the pancreas and revealed that EGFR‐depleted BKC mice (E1KO;BKC) had increased body weights, indicating a better physical condition at the age of 2 and 12 months compared to BKC mice (Fig. 6A, left panel). 100% of E1KO;BKC mice were still clinically unremarkable at the time of dissection (12 months), indicating a drastically prolonged survival compared to BKC mice (data not shown). Their relative pancreas weights were significantly decreased in 8‐week‐old and increased in 12‐month‐old mice compared to BKC mice (Fig. 6A, right panel), implying a better physical condition. Histology analysis revealed only moderate alterations in E1KO;BKC mice, comprising multiple foci presenting ADM (H&E, Fig. 6B), but with a complete lack of fibrosis, shown by Masson’s trichrome staining to pancreata of BKC mice (Fig. 6B). ADM in 8‐week‐old mice did not occur due to incomplete homologous recombination of the EGFR locus, as observed in Ardito *et al*. (2012) and Navas *et al*. (2012), as we verified this possibility in the negative immunostaining targeting EGFR (Fig. 6E). However, ADM was positive for ERBB2, ERBB3, and ERBB4 (Fig. 6E). Interestingly, Western blot analysis revealed that the lack of EGFR is accompanied by a decrease in phosphorylation and expression of ERBB2, ERBB3, and full‐length ERBB4 (Fig. 6C,D). However, we revealed that the ICD of ERBB4 was highly phosphorylated in the pancreata of E1KO;BKC mice compared to BKC mice indicating compensatory ERBB4 signaling for ADM development upon the loss of EGFR (Fig. 6C,D).

## 4 Discussion

While EGFR is crucial for the development of oncogenic KRAS‐induced PDAC development (Ardito *et al*., 2012; Navas *et al*., 2012), targeting EGFR is beneficial only for a small subset of patients (Moore *et al*., 2007). Since EGFR is only one member of a family of four receptor tyrosine kinases, either working autonomously or as partner of ERBB2, ERBB3, or ERBB4, we assumed that the remaining ERBB receptors may have been underestimated in recent years and could also play an important role in PDAC, particularly, because ERBB ligands specific for ERBB4 have been implicated in PDAC development or progression (Chaturvedi *et al*., 2007; Ito *et al*., 2001; Ray *et al*., 2014; Zhu *et al*., 2000). To assess the role of ERBB ligands in PDAC, we deleted BTC in the KC mouse model and revealed that the initiation and progression of PDAC was decelerated. A 100% rate of viable B^−/−^KC mice at the age of 12 months also suggests increased survival rates. The benefits of BTC deletion might stem from decreased EGFR expression and phosphorylation levels observed in B^−/−^KC pancreata at 8 weeks and decreased EGFR phosphorylation at 12 months. It was quite unexpected that the deletion of a single ERBB ligand can modulate EGFR regulation and PDAC progression to such an extent, as in human and in murine PDAC almost all seven EGFR ligands are expressed abundantly and could arguably compensate the loss of one single ligand. At the age of 8 weeks, BKC pancreata presented PDAC and desmoplasia and BKC mice displayed a decrease in the median survival of almost 9 months compared to KC mice. Our study shows that besides TGFA (Siveke *et al*., 2007) and HBEGF (Ray *et al*., 2014), BTC is also highly involved in PDAC development and progression. KC mice have a long latency to develop tumors and although all pancreatic cells are equipped with mutated KRAS, only a subset of cells develops lesions and, at low frequency, invasive PDAC (Hingorani *et al*., 2003). Numerous studies suggest that the additional loss of tumor suppressors (Hingorani *et al*., 2005) or that other secondary events, *f*or *example,* pancreatitis (Carriere *et al*., 2009) or incidences increasing KRAS^G12D^ activity above a certain threshold (Ji *et al*., 2009) are required to initiate tumorigenesis. BTC could be such a factor which enhances RAS activation, thereby possibly accelerating the onset and progression of PDAC. Although BTC activates EGFR, ERBB2, and ERBB4, the increase in RAS activity is mediated by EGFR only, indicating a dependency of RAS activity on EGFR‐homodimerization instead of heterodimerization with ERBB2 or ERBB4. It is not surprising that EGFR is a crucial player in KRAS‐mediated tumorigenesis, since it was previously shown that mice do not develop oncogenic KRAS‐driven PDAC in an EGFR‐depleted background (Ardito *et al*., 2012; Navas *et al*., 2012). EGFR is crucial for acinar cells to transdifferentiate into duct cells. The EGFR ligands TGFA and HBEGF have been previously implicated in this process (Ray *et al*., 2014). BTC has a differentiation potential in many other cell types (Li *et al*., 2005; Mashima *et al*., 1996; Paz *et al*., 2011; Watada *et al*., 1996; Yoshida *et al*., 2002), and we found that BTC is also able to transdifferentiate wild‐type acinar cells into duct cells. However, BTC transgenic mice never developed ADM at any age. Thus, we consider BTC is not an oncoprotein *per se*; however, it seems to act primarily as a trigger for tumorigenesis in the examined mouse models.

While the importance of EGFR in PDAC is well‐established, the role of ERBB2 and ERBB4 is discussed controversially. *ERBB2* mRNA was reported to be expressed in 100% of PDAC samples with increased protein expression compared to normal pancreas (Kolb *et al*., 2007), but another study reported overexpression of ERBB2 in PDAC only at low frequencies (Yan *et al*., 2014). Although *ERBB2* amplification was not associated with the outcome of PDAC in a meta‐analysis (Li *et al*., 2016), another study correlated the overexpression of ERBB2 with an aggressive phenotype (Thybusch‐Bernhardt *et al*., 2001). However, to our knowledge, no functional studies regarding ERBB2 in PDAC exist, and attempts to target ERBB2 were rather disappointing (Harder *et al*., 2012). The deletion of *Erbb2* in BKC mice resulted in a significantly prolonged survival, which can be explained by decelerated PDAC development. Since ERBB2 heterodimerizes with EGFR, it is not surprising that ERBB2 depletion has a beneficial effect in BKC mice. The delay in PDAC progression might be explained by a decrease in EGFR and SAPK signaling. SAPK activity was decreased in ERBB2‐depleted BKC mice, albeit it cannot be associated with decreased apoptosis rates, since cleaved caspase‐3 positivity rather indicated enhanced apoptosis compared to BKC mice. Possibly, decreased SAPK signaling acts via transcriptional regulation rather than by regulating apoptosis.

The ambivalence of ERBB4 in PDAC, as in many other cancers, has emerged from studies assessing tumor‐suppressive functions for the receptor, in that ERBB4 expression was low in PDAC tissues and hPaCaCells (Kolb *et al*., 2007) and decreased in nonmetastatic tumors (Graber *et al*., 1999). Other studies showed that a constitutively active ERBB4 homodimer mutant inhibited colony formation in a pancreatic cancer cell line (Mill *et al*., 2011), and classified ERBB4 as a potential oncogene, after revealing enhanced anchorage‐independent growth of hPaCaCells by the stimulation of ectopic ERBB4 expression (Mill *et al*., 2011). We also attribute ERBB4 an oncogenic function, since its knockout prolonged the survival of BKC mice enormously. The majority of BKC mice lacking ERBB4 showed histopathological pancreatic lesion patterns comparable to BKC mice, which is not in line with the survival outcome. ERBB4‐depleted BKC mice revealed decreased EGFR activity, MAPK‐, and SAPK‐signaling. Decreased MAPK activation is not simply explained by decreased EGFR signaling, since this pathway was not affected by the loss of ERBB2, where we also detected reduced EGFR activity. Thus, these changes must be specific to ERBB4 depletion. Considering that E4KO;BKC mice did not show a reduction in RAS activity, it is surprising that they show reduced MAPK signaling, which acts downstream of RAS. The ERBB4 depletion might result in a considerable change in the downstream targets of RAS. The reduction in MAPK signaling might result in a reduced transcriptional activity of its target genes, thereby acting in an antitumorigenic way. In 1‐week‐old mice, we detected enhanced EGFR and MAPK activity in ERBB4‐depleted BKC mice, indicating that EGFR/MAPK is involved in PDAC induction rather than in PDAC progression. This also supports the thesis of EGFR‐dependency in tumorigenisis in KC mice, but the compensatory mechanism indicates a role for ERBB4 in PDAC initiation, too. It furthermore suggests the causality of EGFR and MAPK activity in an ERBB4‐dependent manner. Possibly, MAPK signaling is preferably regulated by EGFR/ERBB4 heterodimers, independent of ERBB2. Although EGFR signaling was decreased, apparent histopathologic changes were not detected. Increased ERBB3 activation possibly compensates, in part, the loss of EGFR/ERBB4 signaling.

The deletion of EGFR resulted in a nearly complete reversion of the BKC phenotype. However, in 8‐week‐old BKC mice lacking EGFR, ADM lesions were observed multifocally and were verified to be EGFR‐negative, but ERBB2‐, ERBB3‐, and ERBB4‐positive, indicating that, when the receptor family is challenged, other receptors take over to induce ADM. Interestingly, the lack of EGFR was accompanied by the downregulation of ERBB2, ERBB3, and full‐length ERBB4, but it induced ERBB4‐ICD signaling, indicating a compensatory mechanism upon the loss of EGFR. Possibly, the ERBB4‐ICD is responsible for ADM development in EGFR‐depleted BKC mice, since all remaining receptors expressed in the ADM lesions were less phosphorylated. These findings are in line with a study that associated BTC with resistance to EGFR treatment in breast cancer cell lines (Kong *et al*., 2008). The treatment with tyrosine kinase inhibitors led to acute BTC expression, induction of ERBB2/ERBB4 dimers, and ERBB4 cleavage, thereby evading EGFR inhibition and reactivating EGFR‐mediated signaling cascades. This is partially in line with our results regarding ERBB4 activation and ERBB4‐RIP upon BTC activation in an EGFR‐depleted PDAC mouse model. This compensational mechanism of the ERBB family could possibly play a role in the resistance of an EGFR inhibitor therapy in PDAC patients and in attenuated PDAC development in EGFR/TP53‐depleted KC mice (Ardito *et al*., 2012; Navas *et al*., 2012).

## 5 Conclusions

We have shown that the depletion of BTC ameliorates the outcome of PDAC and, conversely, that BTC overexpression deteriorates PDAC prognosis in KC mice. BTC involves not only EGFR activation, but it also harnesses its 'partners in crime' to induce accelerated PDAC development and progression. BTC enhances RAS activity, thereby potentially transforming acinar to duct cells. The partial disease rescue upon loss of ERBB2 and ERBB4 implies that both receptors are potent oncogenes in PDAC. Especially, enhanced ERBB4‐ICD activation upon EGFR depletion points to a compensatory behavior of the ERBB family, thus emphasizing their role as co‐targets for combinatorial EGFR‐targeted therapies. Our data endorse that the pan‐ERBB inhibitor dacomitinib exhibits stronger antitumor effects than conventional single‐receptor targeting of PDAC cells (Momeny *et al*., 2019). We suggest that targeting the complete ERBB family, instead of a single receptor, might be promising for future custom PDAC therapy development.

## Conflict of interest

The authors declare no conflict of interest.

## Author contributions

MD and KH acquired and interpreted the data. HA, MD, KH, ML, and AB analyzed and interpreted the data. HA, MD, KH, ML, and MRS prepared the manuscript. RMS critically revised the manuscript.

## Supporting information


**Fig. S1.** Generation and verification of the BTC knockout mouse.

**Fig. S2.** Activation of EGFR in pancreata of 1‐week‐old mice.

**Fig. S3.** Change of BKC pancreata upon ERBB2 depletion in 8‐week‐old mice.

**Table S1.** Primers employed for genotyping PCR.
**Table S2.** Antibodies employed for immunohistochemistry.
**Table S3.** Antibodies employed for Western blot.
