# Supplementary material for: Unraveling ERBB network dynamics upon betacellulin signaling in pancreatic ductal adenocarcinoma in mice
Source: Mol Oncol. 2020 May 18;14(8):1653–69. doi: 10.1002/1878-0261.12699 (PMC7400790; doi:10.1002/1878-0261.12699)
Supplement: Supplementary file 1 — Fig. S1. Generation and verification of the BTC knockout mouse. [file MOL2-14-1653-s001.pdf]

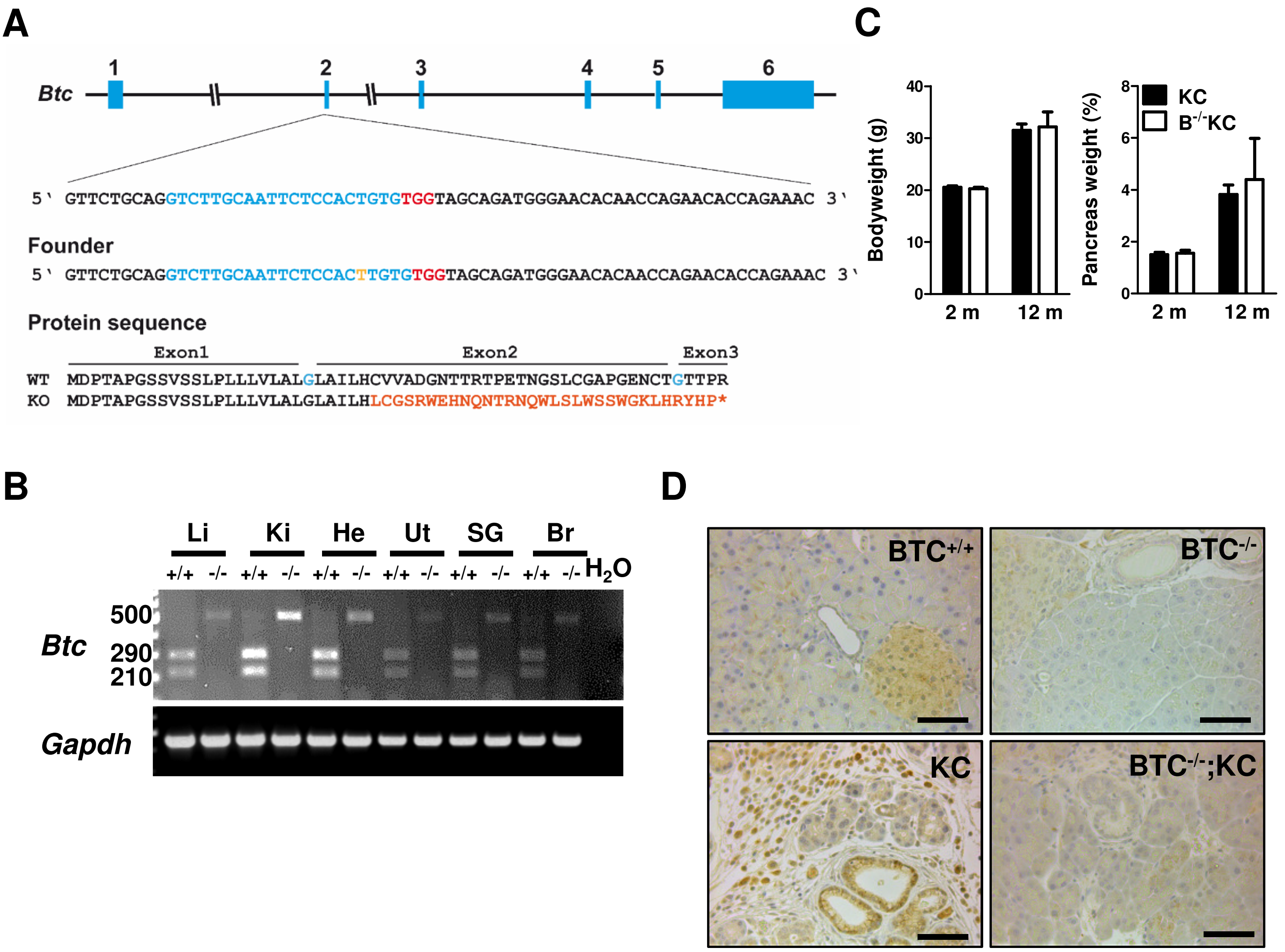

Supplementary Figure S1. Generation and verification of the BTC knockout mouse. (A) Partial DNA sequence of *Btc* exon 2. The sgRNA binding site is indicated in blue, the protospacer adjacent motif (PAM) in red. Insertions (orange) of 1 bp lead a shift of reading frame. Partial amino acid sequences encoded by the WT and mutant *Btc* alleles. WT BTC aa sequence in black (aa coded by adjacent non-symmetrical exons in blue), missense aa sequence in red, and the premature termination codon as asterisk. WT=wildtype. KO=knockout. (B) Verification of the frame shift by reverse-transcriptase PCR of Li (liver), Ki (kidney), He (heart), UT (uterus), SG (salivary gland), Br (brain) after the restriction of the 500 bp amplicon with *PflM*I into fragments of 290 and 210 bp, respectively. The successful cleavage by *PflM*I indicates wildtype *Btc* (*Btc*$^{+/+}$), while the lack of cleavage indicates the successful frame shift introduced by CRISPR/Cas9 and hence the BTC knockout (*Btc*$^{-/-}$). H_20 served as negative control, *Gapdh* as control PCR. (C) The crossing of BTC$^{-/-}$ mice into the KC background showed no change of body or pancreatic weights at the ages of 2 or 12 months (m), respectively. (D) Immunohistochemistry targeting BTC in the pancreas of wildtype mice (BTC$^{+/+}$) marking the islets of Langerhans and the cell membrane of acinar cells, BTC knockout mice (BTC$^{-/-}$) presenting no signal for BTC staining. KC mice express BTC in premalignant lesions and BTC$^{-/-}$;KC mice are immuno-negative for BTC. Scale bars: 100 µm.
