# Supplementary material for: Unraveling ERBB network dynamics upon betacellulin signaling in pancreatic ductal adenocarcinoma in mice
Source: Mol Oncol. 2020 May 18;14(8):1653–69. doi: 10.1002/1878-0261.12699 (PMC7400790; doi:10.1002/1878-0261.12699)
Supplement: Supplementary file 2 — Fig. S2. Activation of EGFR in pancreata of 1‐week‐old mice. [file MOL2-14-1653-s002.pdf]

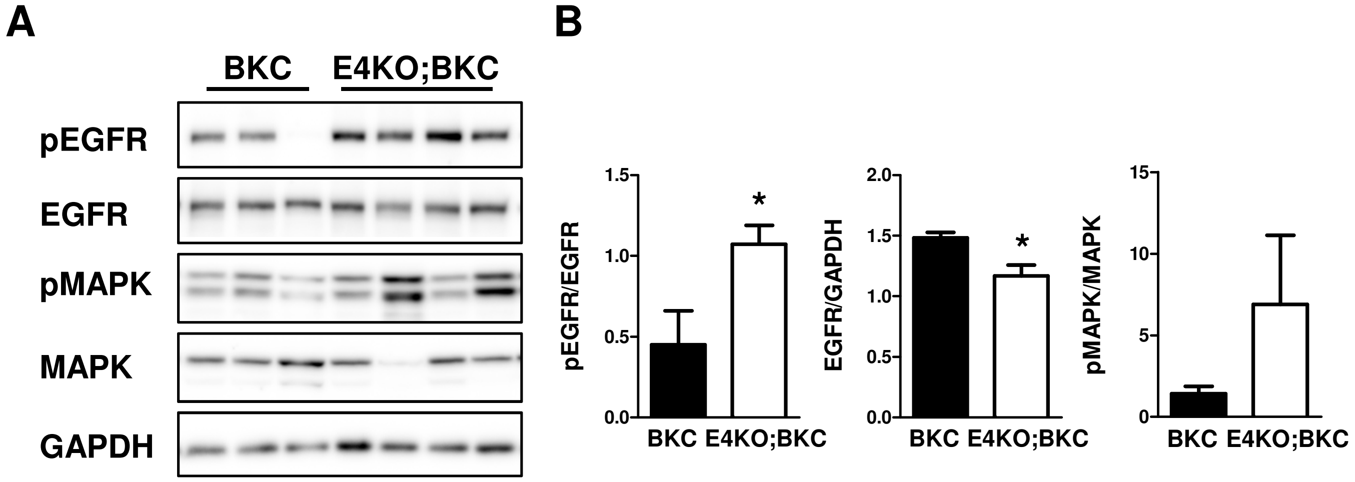

Supplementary Figure S2. Activation of EGFR in pancreata of 1-week-old mice. (A) Western blot and (B) corresponding densitometrical analyses of pancreata of 1-week-old ERBB4 KO;BKC mice depict enhanced EGFR activation and decreased EGFR expression compared to age-matched BKC mice. GAPDH served as reference protein. Data were analyzed by Student's *t*-test, **P*<0.05.
