# Supplementary material for: Unraveling ERBB network dynamics upon betacellulin signaling in pancreatic ductal adenocarcinoma in mice
Source: Mol Oncol. 2020 May 18;14(8):1653–69. doi: 10.1002/1878-0261.12699 (PMC7400790; doi:10.1002/1878-0261.12699)
Supplement: Supplementary file 3 — Fig. S3. Change of BKC pancreata upon ERBB2 depletion in 8‐week‐old mice. [file MOL2-14-1653-s003.pdf]

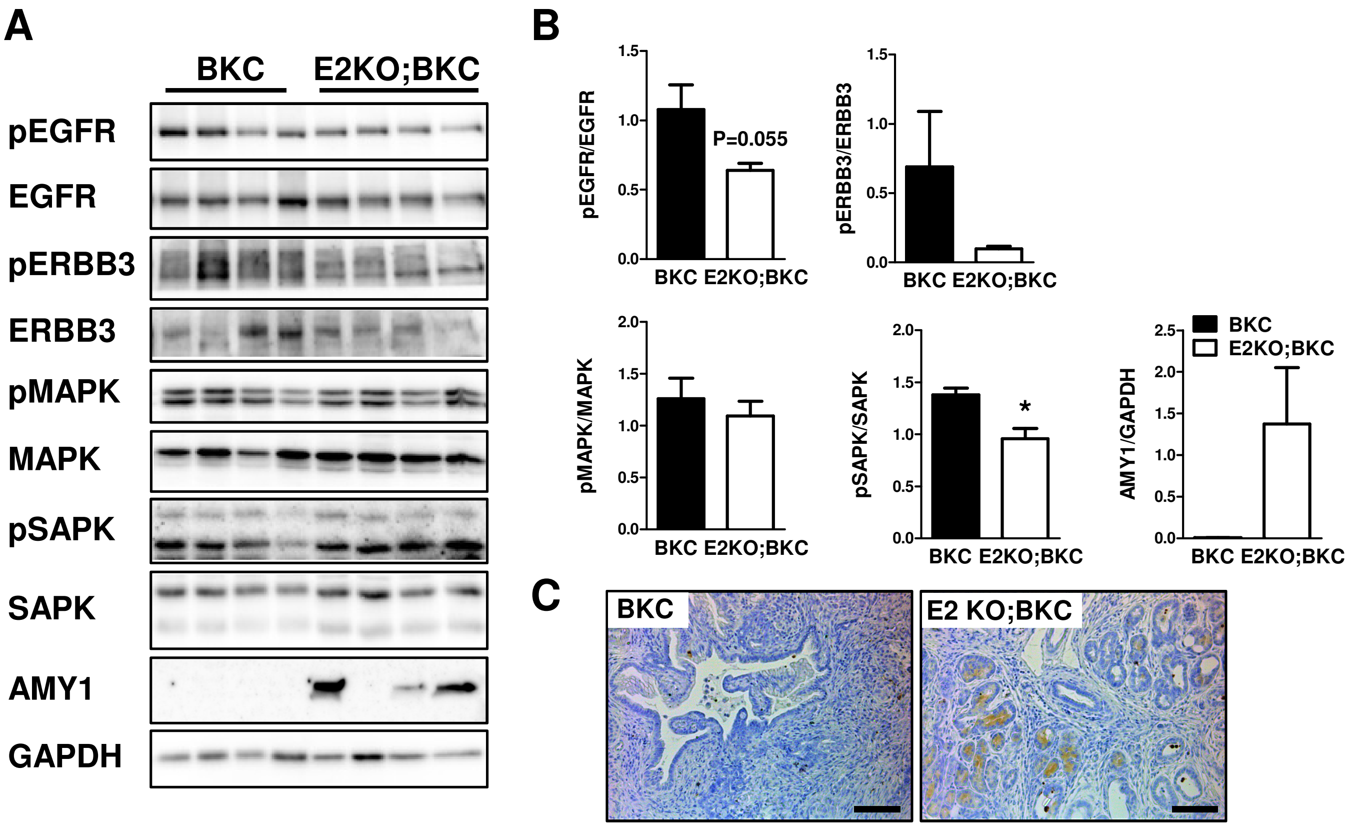

Supplementary Figure S3. Change of BKC pancreata upon ERBB2 depletion in 8-week-old mice. (A) Western blots and (B) corresponding densitometrcal analyses of BKC pancreata compared to E2KO;BKC pancreata revealed a significantly decreased SAPK activation and remaining amylase (AMY1) levels in E2KO;BKC mice. Data were analyzed by Student's *t*-test, *$P<0.05$. (C) Immunohistochemical detection of cleaved caspase 3 in pancreata of BKC mice lacking ERBB2 compared to BKC mice demonstrates the activation of apoptosis in acinar clusters upon the loss of ERBB2. Scale bars: 100 µm.
