# Supplementary material for: Unraveling ERBB network dynamics upon betacellulin signaling in pancreatic ductal adenocarcinoma in mice
Source: Mol Oncol. 2020 May 18;14(8):1653–69. doi: 10.1002/1878-0261.12699 (PMC7400790; doi:10.1002/1878-0261.12699)
Supplement: Supplementary file 4 — Table S1. Primers employed for genotyping PCR. Table S2. Antibodies employed for immunohistochemistry. Table S3. Antibodies employed for Western blot. [file MOL2-14-1653-s004.docx]

**Supplementary Table 1.** Primers employed for genotyping PCR.

| Target | Primer | Sequence |
| --- | --- | --- |
| *Egfr^tm1Dwt^* | Forward Primer  Reverse Primer | 5’-ctttggagaacctgcagatc-3’  5’-ctgctactggctcaagtttc-3’ |
| *Erbb2^fl/fl^* | Forward Primer  Reverse Primer | 5’-GACAAGGTTTCTCTGTGTAGC-3’  5’-GTGAGTTACAGACCAAGCCC-3’ |
| *Erbb4^fl/fl^* | Forward Primer  Reverse Primer1  Reverse Primer2 | 5’-CAAATGCTCTCTCTGTTCTTTGTGTCTG-3’  5’-TTTTGCCAAGTTCTAATTCCATCAGAAGC-3’  5’-TATTGTGTTCATCTATCATTGCAACCCAG-3’ |
| *Kras^fl/fl^* | Forward Primer1  Forward Primer2  Reverse Primer | 5’-CCATGGCTTGAGTAAGTCTGC-3’  5’-CACCAGCTTCGGCTTCCTATT-3’  5’-AGCTAATGGCTCTCAAAGGAATGTA-3’ |
| *Ptf1a^tm1(cre)Hnak^* | Forward Primer  Reverse Primer | 5’-AATCGCCATCTTCCAGCAG-3’  5’-GATCGCTGCCAGGATATACG-3’ |
| *Btc-tg* | Forward Primer  Reverse Primer | 5’-CTACAGCTCCTGGGCAACGTG-3’  5’-ATGAGTCAGGTCTTTTGTAGCTTG-3’ |
| *BTC^-/-^* (genotyping) | Forward Primer  Reverse Primer | 5’-GCTTACATGACTTCTAGCCATG-3’  5’-GCTAATCATGGAGGCACAGAAA-3’ |
| *BTC^-/-^* (RT-PCR) | Forward Primer  Reverse Primer | 5’-GTTCTGGGTGACACTGGAGC-3’  5’-CCTTTCTCACAGATGCAGGAG-3’ |
| *Gapdh* | Forward Primer  Reverse Primer | 5’-GTGGAAGGGCTCATGACCAC-3’  5’-GCCCACAGCCTTGGCAGCA-3’ |

**Supplementary Table 2.** Antibodies employed for immunohistochemistry.

| **Antigen** | **Antibody** | **Host** | **Dilution** |
| --- | --- | --- | --- |
| mBTC | R&D Systems, Minneapolis, MN, USA #AF1025 | Goat | 1:500 |
| hBTC | R&D Systems, AF261NA | Goat | 1:300 |
| TGFA | Merck, Darmstadt, Germany, GF10 | Mouse | 5 µg/ml |
| AREG | R&D Systems, MAB989 | Mouse | 5 µg/ml |
| EREG | R&D Systems, AF1068 | Goat | 5 µg/ml |
| ACTA2 | Proteintech, Manchester, UK, #14395-1-AP | Rabbit | 1:200 |
| mEGFR | R&D Systems, AG1280 | Goat | 5 µg/ml |
| hEGFR | R&D Sytems #AF231 | Goat | 1:800 |
| hERBB2 | Cell Signaling #4250 | Rabbit | 1:800 |
| hERBB3 | Cell Signaling #12708 | Rabbit | 1:500 |
| hERBB4 | Proteintech #19941-1-AP | Rabbit | 1:400 |
| Cleaved Caspase 3 | Cell Signaling #9661 | Rabbit | 1:200 |
| Donkey α Goat | R&D Systems, #HAF109 | Donkey | 1:200 |
| SignalStain® Boost IHC Detection Reagent | Cell Signaling #8114 |  | Ready to use |

**Supplementary Table 3.** Antibodies employed for Western blot. All antibodies from Cell Signaling and mBTC (R&D) were diluted in 5% BSA in TBS‑T, while all Santa Cruz antibodies were diluted in 5% milk in TBS‑T.

| **Antigen** | **Antibody** | **Host** | **Dilution** |
| --- | --- | --- | --- |
| p-EGFR (Tyr1068) | Cell Signaling, Boston, USA #3777 | Rabbit | 1:1000 |
| p-ERBB2 (Tyr877) | Cell Signaling #2241 | Rabbit | 1:1000 |
| p-ERBB3 (Tyr1289) | Cell Signaling #4791 | Rabbit | 1:1000 |
| p-ERBB4 (Tyr 1258) | Abcam, Cambridge, UK ab76132 | Rabbit | 1:1000 |
| EGFR | Santa Cruz, Heidelberg, Germany #03 | Rabbit | 1:500 |
| ERBB2 | Santa Cruz #284 | Rabbit | 1:500 |
| ERBB3 | Santa Cruz #285 | Rabbit | 1:500 |
| ERBB4 | Santa Cruz #283 | Rabbit | 1:500 |
| p-SAPK | Cell Signaling #9251 | Rabbit | 1:1000 |
| SAPK | Cell Signaling #9252 | Rabbit | 1:1000 |
| p-p44/42 MAPK (Thr202/Tyr204) | Cell Signaling #4370 | Rabbit | 1:1000 |
| p44/42 MAPK | Cell Signaling #9102 | Rabbit | 1:1000 |
| CDH1 | Cell Signaling #14472 | Mouse | 1:1000 |
| mRAS | Cell Signaling #8832 | Mouse | 1:200 |
| mBTC | R&D Systems #AF1025 | Goat | 1:2000 |
| TUBA1A | Cell Signaling, # 2125 | Rabbit | 1:5000 |
| GAPDH | Cell Signaling, #2118 | Rabbit | 1:5000 |
| Goat α Rabbit | Cell Signaling, #7074 | Goat | 1:2500 |
| Rabbit α Mouse | Cell Signaling, #7076 | Rabbit | 1:2000 |
